# Association between the pri-miR-26a-1 rs7372209 C>T polymorphism and cancer susceptibility: multivariate analysis and trial sequential analysis

**DOI:** 10.18632/aging.103696

**Published:** 2020-10-14

**Authors:** Yuan-Yuan Hu, Guang-Bin Jiang, Ya-Feng Song, Ai-Ling Zhan, Cai Deng, Yu-Ming Niu, Lan Zhou, Qi-Wen Duan

**Affiliations:** 1 Department of Stomatology, Department of Clinical Oncology, Hubei Key Laboratory of Embryonic Stem Cell Research, Taihe Hospital, Hubei University of Medicine, Shiyan 442000, China; 2 Department of Radiology, Suizhou Hospital, Hubei University of Medicine, Suizhou Central Hospital, Suizhou 441300, China; 3 The Personnel Section, Taihe Hospital, Hubei University of Medicine, Shiyan 442000, China; 4 Department of Neurology, Taihe Hospital, Hubei University of Medicine, Shiyan 442000, China; 5 Department of Anesthesiology, Central Hospital of Shanghai Songjiang District, Shanghai 201600, China

**Keywords:** pri-miR-26a-1, cancer, polymorphism, multivariate analysis, trial sequential analysis

## Abstract

MiR-26 has been suggested to play a tumor-suppressive role in cancer development, which could be influenced by the mutate pri-miR-26ª-1. Molecular epidemiological studies have demonstrated some inconsistent associations between pri-miR-26ª-1 rs7372209 C>T polymorphism and cancer risk. We therefore performed this meta-analysis with multivariate statistic method to comprehensively evaluate the associations between rs7372209 C>T polymorphism and cancer risk. Eleven publications involving 6,709 patients and 6,514 controls were identified. Multivariate analysis indicated that the over-dominant genetic model was most likely. Pooled results indicated no significant association in the overall population (CC+TT vs. CT: OR=1.08, 95%CI=0.96-1.22, *P*=0.20, *I*^2^=54.4%), as well as the subgroup analysis according to ethnicity, control source, tumor locations, and HWE status of controls. In addition, heterogeneity, accumulative, sensitivity analysis, publication bias and trial sequential analysis (TSA) were conducted to test the statistical power. Overall, our results indicated that the pri-miR-26a-1 rs7372209 C>T polymorphism may not be a potential risk for cancer development.

## INTRODUCTION

Cancer is a leading cause of death worldwide [1]. In 2012, approximately 14.1 million people presented with cancer, and 8.2 million people died from this disease globally. [2]. In 2018, the number of new cancer cases and cancer-related deaths increased rapidly to 18.1 and 9.6 million, respectively [3]. In China, more than 4,292,000 new cancer cases and 2,814,000 deaths were recorded in 2015. To date, cancer is considered the most common cause of death with increasing incidence and mortality worldwide [4]. Surgery, radiotherapy, chemotherapy, and other types of treatments have been widely applied for the treatment of this condition. However, all treatments have harmful side effects. Damage in the tissues and organs will result in some dysfunctions and will significantly reduce quality of life. Moreover, cancer care and treatment can cause heavy economic and mental burden to the society and the patients’ families. Several studies have been conducted to explore the etiology and pathogenesis of cancer development in the past decades. However, the underlying mechanism and the susceptibility of individuals with this disease remain poorly understood. Environmental factors, unhealthy lifestyle habits, viral infections and chronic inflammation are associated with cancer occurs.

The aberrant expression of related genes in a cell causes abnormalities in cell proliferation and cancer development. The microRNA family comprises important small non-coding RNA molecules that have a length of 21-25 nucleotides and are characterized by double-stranded structures [5, 6], which originate from the primary transcripts (pri-miRNAs) via continuous maturation procedures.

MicroRNA (miRNA) can regulate their posttranscriptional repression by binding to 3′-untranslated region (3-UTR) of the target gene mRNAs with imperfect complementary sequences [7]. The abnormally expressed microRNA can act as proto-oncogene and anti-oncogene via various cellular signaling pathways based on several reports [8, 9]. MiRNA-26a is a new microRNA that plays a tumor-suppressive role during cell cycle by inhibiting cancer cell proliferation, invasion, and metastasis [10, 11]. The expression of miRNA-26a in cancer cells is significantly reduced compared with that in normal tissues, and its expression levels are significantly associated with tumor size, pathologic differentiation, clinical stage, and overall prognosis [12, 13].

Gene mutation in miRNA or pri-miRNA can affect miRNA function via several different biosynthetic pathways. Single nucleotide polymorphism (SNP) is one of the most common type of gene mutation, and the SNPs in pri-miRNA genes can change spatial structure, affect the miRNA–mRNA interaction network, activate the aberrant expression of target genes and increase the risk of cancer. For pri-miR-26a-1, rs7372209 C>T is the most common locus that has attracted more attentions. In 2008, Yang et al. published the first case-control study of Chinese population and results did not show any significant association between the pri-miR-26a-1 rs7372209 C>T polymorphism and bladder cancer [14]. Subsequently, numerous epidemiological studies have been performed to examine the relationship between the pri-miR-26a-1 rs7372209 C>T polymorphism and the risk of cancer. However, the results were contrasting. Therefore, this meta-analysis aimed to conduct a precise and comprehensive assessment of the association between the pri-miR-26a-1 rs7372209 C>T polymorphism and the risk of cancer.

## RESULTS

### Study characteristics

**Figure 1:**
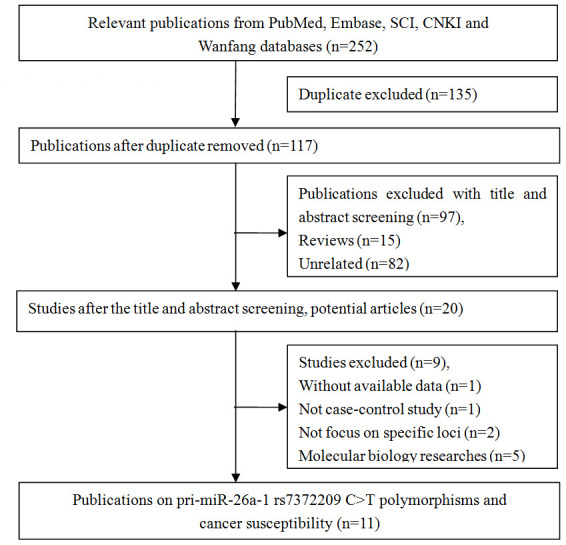
**Flow diagram of the study selection process.**

In total, 252 articles were retrieved via a system search. Among these studies, 135 were excluded during the first step of article duplication and 97 during the abstract and full-text review (Figure 1). Finally, 11 articles and 12 independent case-control studies, which included 6,709 patients with cancer and 6,514 controls, met our inclusion criteria [14–24]. The selection process is depicted in Figure 1. There were 9 case-control studies with 5,426 cases and 4,788 controls in the Asian populations (Chinese) [15, 16, 18–24], 1 case-control study with 362 cases and 578 controls in the African population [17], and 1 case-control study with 193 cases and 420 controls in the mixed population [17]. Four studies used the TaqMan method [16, 17, 24], two studies used the MassARRAY method [20, 23], two studies used the polymerase chain reaction-ligase detection reaction method [18, 21], and the remaining studies used other methods (including MALDI-TOF mass spectrometry,, SNaPshot, and Illumina) [15, 19, 22]. Moreover, four studies focused on esophageal squamous cell carcinoma [15, 17, 19], two studies on lung cancer [16, 22], two studies on colorectal cancer [21, 23], and one study on cervical cancer [18], breast cancer [20], and oral cancer [24]. The genotype distributions of the control groups in three studies deviated from the HWE [15, 17, 22], and the remaining studies were all satisfied with HWE status. In seven studies, the NOS score was greater than 8. The other two studies had 8 points and one study was 7 points. All data about the included studies are presented in Table 1.

**Table 1:** Characteristics of included studies on pri-miR-26a-1 rs7372209 C>T polymorphism and cancer risk.

**First author**	**Year**	**Country /Region**	**Racial**	**Source of controls**	**Case**	**Control**	**Genotype distribution**							**Genotyping methods**	***P* for HWE^a^**	***MAF in control***	**Tumor type**	**NOS**
							**Case**				**Control**							
							**CC**	**CT**	**TT**		**CC**	**CT**	**TT**					
Yang-A	2008	US	European	PB	728	728	362	324	42		378	288	62	SNPlex assay	0.50	0.28	BLC	10
Wei	2013	China	Asian	HB	380	380	187	164	29		178	178	24	MALDI-TOF MS	0.02	0.30	ESCC	8
Wang-1	2013	South Africa	African	PB	362	578	350	12	0		546	32	0	TaqMan	0.49	0.03	ESCC	9
Wang-2	2013	South Africa	Mixed	PB	193	420	166	26	1		307	110	3	TaqMan	0.04	0.14	ESCC	8
Li	2014	China	Asian	HB	648	672	242	319	87		293	315	64	TaqMan	0.12	0.33	LC	8
Xiong	2014	China	Asian	HB	417	103	221	167	29		57	36	10	PCR–LDR	0.23	0.27	CC	9
Zhang-A	2014	China	Asian	PB	1109	1275	541	454	114		628	538	109	SNaPshot	0.68	0.30	ESCC	10
Zhang-B	2015	China	Asian	PB	384	192	210	142	30		99	74	18	Sequenom	0.45	0.29	BRC	11
Liu	2016	China	Asian	HB	721	626	391	268	59		334	252	40	PCR-LDR	0.41	0.27	CRC	8
Yin	2016	China	Asian	HB	268	266	137	111	20		125	129	12	Illumina	<0.01	0.29	LC	7
Ying	2016	China	Asian	HB	1344	1079	737	514	93		582	432	65	Sequenom	0.20	0.26	CRC	9
Yang-B	2017	China	Asian	HB	160	196	80	65	15		90	80	26	TaqMan	0.23	0.34	OC	9

^a^HWE in control

BLC: Bladder cancer; ESCC: Esophageal Squamous Cell Carcinoma; LC: lung cancer; CC: Cervical cancer; BRC: breast cancer; CRC: colorectal cancer; OC: oral cancer; MAF: Minor allele frequency in control group; PB: Population-based HB: Hospital-based; PCR-LDR: Polymerase chain reaction -ligase detection reaction.

### Quantitative analysis

**Figure 2:**
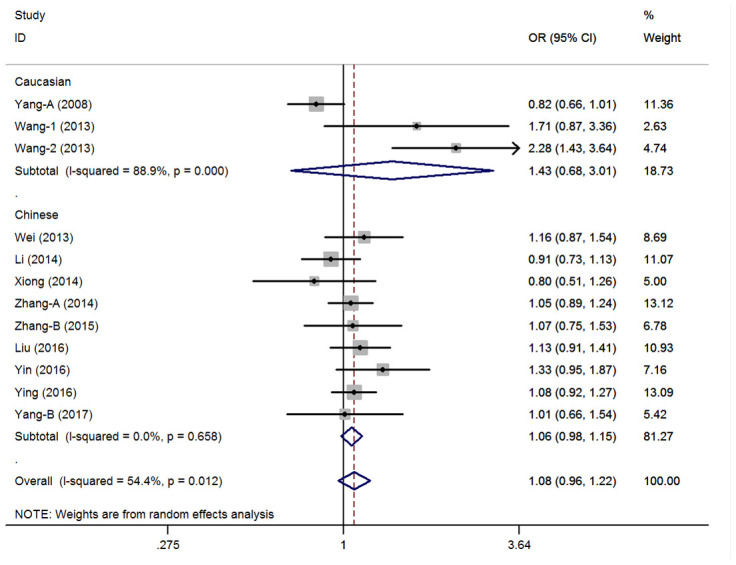
**Statistical analysis of the association between pri-miR-26a-1 rs7372209 C>T polymorphism and cancer risk in over-dominant model.**

The estimated λ=-0.81 (95%CI=-3.85-2.24) and less than zero. Hence, the over-dominant genetic model could be used in the general population (CC+TT vs. CT: OR=1.08, 95%CI=0.96-1.22, *P*=0.20, *I*^2^=54.4%) (Figure 2, Supplementary Table 1). Heterogeneity was observed, and a meta-regression analysis was conducted. Results showed that the HWE status might have caused the heterogeneity (t=2.37, *P*=0.04). Then, three studies that did not satisfy the HWE were excluded and the pooled analysis didn’t present any significant result with the remaining nine case-control studies (CC+TT vs. CT: OR=1.01, 95%CI=0.93-1.09, *P*=0.84, *I*^2^=21.5%) (Supplementary Table 1). Moreover, the subgroup analysis revealed other similar negative associations according to ethnicity, control design, and tumor locations. Then, the remaining genetic models were assessed via univariate analyses, and no significant association was found between the pri-miR-26a-1 rs7372209 C>T polymorphism and the risk of cancer in the whole population (Supplementary Table 1). However, the following subgroup analysis conducted according to ethnicity revealed that the TT genotype indicated a slightly higher risk for cancer development in the Chinese population (TT vs. CC: OR=1.19, 95%CI=1.02-1.38, *P*=0.03, *I*^2^=16.0%; TT vs. CC+CT: OR=1.19, 95%CI=1.03-1.38, *P*=0.02, *I*^2^=0%) and other stratified analyses of hospital based control group and lung cancer subgroup (Supplementary Table 1).

### Cumulative and sensitivity analyses

**Figure 3:**
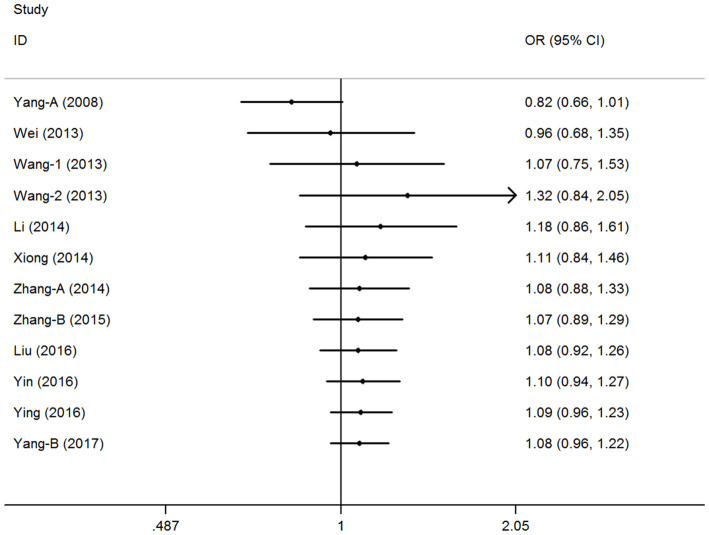
**Cumulative meta-analyses according to publication year in over-dominant model of pri-miR-26a-1 rs7372209 C>T polymorphism.**

**Figure 4:**
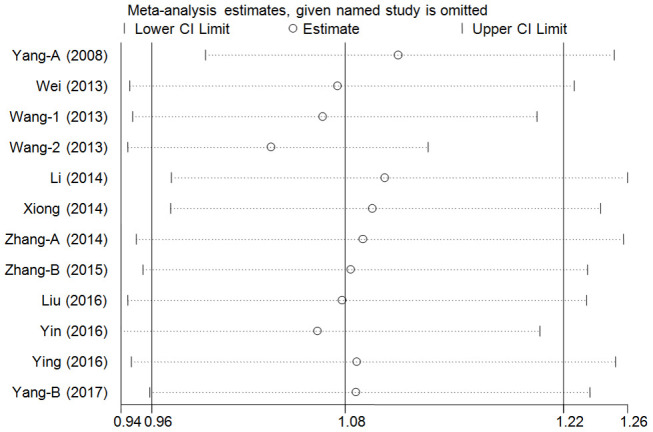
**Sensitivity analysis through deleting each study to reflect the influence of the individual dataset to the pooled ORs in over-dominant model of pri-miR-26a-1 rs7372209 C>T polymorphism.**

A cumulative analysis of the publication date was conducted in the over-dominant model, and results showed that the pooled ORs were not qualitatively affected by the additional studies, indicating that the results were highly stable (Figure 3). Sensitive analyses were conducted by deleting each study, and results showed that the findings were consistent (Figure 4).

### Publication bias

**Figure 5:**
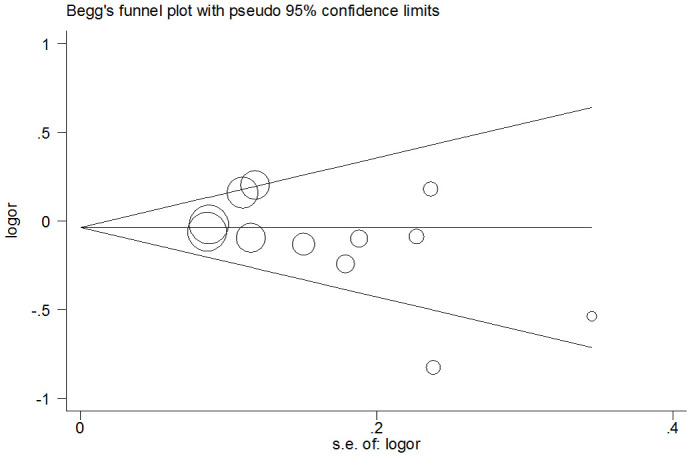
**Funnel plot analysis to detect publication bias for over-dominant model of pri-miR-26a-1 rs7372209 C>T polymorphism.** Circles represent the weight of the studies.

In the over-dominant model, the presence of publication bias was examined using the Begg’s test, and results did not find any asymmetry in the funnel plot. These results were confirmed with the Egger’s test (CC+TT vs. CT: T=1.44, *P*=0.18) (Figure 5).

### Trial sequential analysis

**Figure 6:**
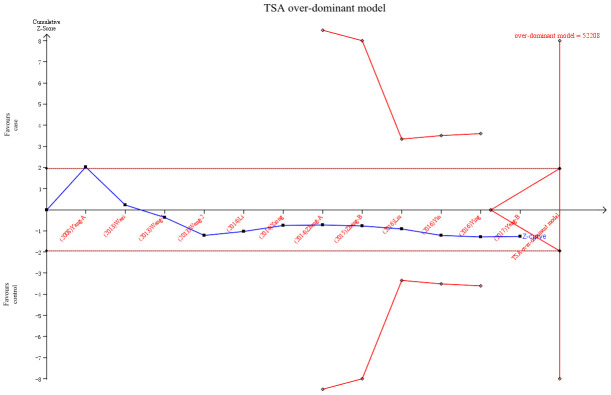
**Trial sequential analysis of pri-miR-26a-1 rs7372209 C>T polymorphism and cancer risk in over-dominant model. The blue line represents the cumulative Z-score of the meta-analysis.** The red straight represent the conventional P=0.05 statistical boundaries.

TSA was conducted in the over-dominant model. Result showed that the cumulative Z-curve (blue line) did not cross the conventional P=0.05 boundary (red straight lines) and did not reach the required information sizes (n=52208). These data indicated that the cumulative evidence on the pri-miR-26a-1 rs7372209 C>T polymorphism was not adequate and more trials were required (Figure 6).

## DISCUSSION

MiRNAs belong to the family of non-coding small RNAs that comprise 22-25 nucleotides, which can regulate the target gene expression via the post-transcriptional pathway by binding to the 3'-UTRs [25]. The miRNA sequences are highly conserved during evolution, and they play a role in multiple physiological or pathological processes, including cell proliferation, differentiation, and apoptosis [26]. The aberrant mutation of pri-miRNAs could change the nucleotide sequence and spatial structure of the corresponding miRNAs, thereby interfering with the normal physiological processes of the cells and consequently leading to the formation and proliferation of abnormal tumor cells [27–30].

Pri-miR-26a-1 is a novel, small RNA that involves several signaling pathways and acts as a tumor suppressor in tumorgenesis and cancer development by binding to Lin28B and Zcchc11 to suppress cancer development and metastasis [31, 32]. The pri-miR-26a-1 gene is located in the human chromosome 3q21.3, and rs7372209 C>T polymorphism is the most important SNP locus in the pri-miR-26a-1 gene, which significantly associated with susceptibility to various types of cancers. Since 2008, numerous case-control studies on rs7372209 C>T polymorphism have been conducted. However, the results were inconsistent.

Yang et al. (2008) first analyzed the frequency of rs7372209 C>T polymorphism in individuals with bladder cancer and indicated less susceptibility in dominant model (OR=1.10, 95% CI=0.89-1.36, *P*=0.38) in US [14]. Thereafter, Wei et al. [15] and Zhang et al. [19] studied the rs7372209 C>T polymorphism to identify its effect on esophageal cancer susceptibility among Chinese and showed that there was no significant difference between the control and case groups in genotypic distribution in 2013 and 2014 separately (for dominant model: OR=0.89, 95% CI=0.67-1.18, *P*=0.42; OR=1.03, 95% CI=0.87-1.21, *P*=0.73), indicating no significant risk for esophageal cancer. In contrast, Wang et al. reported that South Africans with rs7372209 C>T polymorphism had a significantly reduced risk for esophageal squamous cell carcinoma in the additive model (OR=0.47, 95% CI=0.28-0.78, *P*=0.003) and the dominant genetic model (OR=0.44, 95% CI=0.26-0.74, *P*=0.002) in the mixed ancestry group in 2013 [17].

In 2014, Li et al. investigated the association between rs7372209 C>T polymorphism and the risk of lung cancer and found that the subjects with T allele of rs7372209 C>T polymorphism had an increased risk of cancer development (OR=1.27, 95% CI=1.07-1.50, *P*=0.01) [16]. On the contrary, another case-control study conducted by Yin et al. focused on the correlation between rs7372209 C>T polymorphism and lung cancer. This study showed that this variant was not significantly associated with the risk of lung cancer [22]. Then, Xiong et al. conducted a case-control study on the association between rs7372209 C>T polymorphism and the risk of cervical cancer among southern Chinese women. However, this study did not identify any significant relationship between the polymorphic loci and the risk of cervical cancer [18]. In 2015, Zhang et al. evaluated the association between rs7372209 C>T polymorphism and the risk of breast cancer among Chinese women, and results showed that there was no significant association between them [20]. Moreover, Further, Liu et al. [21] and Ying et al. [23] revealed that the rs7372209 C>T polymorphism was not significantly associated with colorectal cancer in 2016. In addition, the protective effect of rs7372209 in the dominant model was observed in advanced-stage oral squamous cell carcinoma in the study of Yang et al. (OR=0.57, 95% CI=0.38-0.87, *P*=0.01) [24].

The above mentioned controversy may be attributed to the following: (1) the populations assessed were of different ethnicities, (2) varying genotype methods could influence outcomes, (3) deviation from HWE could be observed in some studies, and (4) the design and procedure of each research were not similar, thereby reducing the consistency. Hence, we conducted a meta-analysis of 11 publications (12 independent case-control studies) involving 12,223 participants to assess the association between rs7372209 C>T polymorphism and cancer susceptibility.

Based on our knowledge, the etiology and pathogenesis of cancer development remains unclear. Increasing evidences has shown that some miRNAs and the genetic polymorphisms of miRNAs are associated with cancer susceptibility. In the current meta-analysis, we comprehensively summarized data on the relationship between rs7372209 C>T polymorphism and the risk of cancer, and no significant association was found between rs7372209 C>T polymorphism and cancer in the whole population and in the subgroup with the finest genetic model. These results indicated that these genetic polymorphisms may not be the only factor affecting the development of cancer. Furthermore, some univariate analyses revealed that Chinese have increased risks of rs7372209 C>T polymorphism and cancer. The co-dominant and recessive models indicated that the TT mutant homozygote was associated with a 19% increased risk of cancer development in Chinese, but not in Caucasians. These differences were assumed maybe due to the number of relative studies (9 case-control studies on Chinese and 3 case-control studies on Caucasians) between two ethnicities. In general, tumor formation often involves a complex process, during which a variety of factors and proteins participate and of complex signal transduction network. Variations in a single gene and locus might not play a decisive role in tumorigenesis affecting the entire signaling pathway, so did the rs7372209 C>T polymorphism too. Nevertheless, a more scientific statistical method was used to select a better gene model through multiple regressions to analyze the mutation of this polymorphic site and the susceptibility of cancers in this study. Then, the analysis indicated that the over-dominant genetic model could be the most appropriate choice and the results revealed there was no significant association between rs7372209 C>T polymorphism and cancers in the general and subgroup analyses.

To date, this study conducted the first meta-analysis on the association between rs7372209 C>T polymorphism and the risk of cancer. It has some advantages, which were as follows: (1) a more advanced method with multivariate meta analyses was used to select the genetic model; (2) more scientific search strategies and rigorous statistic methodologies were utilized; and (3) sensitivity, accumulation, and meta-regression analyses were conducted to identify the potential interfering factors that can contribute to the inconsistencies in the results; and (4) the TSA was conducted and indicated that the current data were not enough. The current study had some limitations that should be emphasized. First, only one SNP locus was examined in this meta-analysis, and the interaction mechanisms between gene-gene and gene-environment were not assessed due to the limited number of data. Second, heterogeneity was observed in the included studies, which might affect the current results. However, it was partially alleviated in the subsequent stratified analysis, such as that conducted in the Chinese population and the hospital-control design and colorectal cancer groups. Third, almost all studies included from Asians, thereby limiting the application of our results in the general population. Fourth, all summarized results were based on published papers, which might have distorted the actual effect due to publication bias even if there was no significant publication bias found using both Egger’s test and Begg’s funnel plot.

In summary, the pri-miR-26a-1 rs7372209 C>T polymorphism may not be an independent risk factor for tumorigenesis and the development of cancer. Owing to the insufficient sample size, more high-quality studies with a large sample size must be conducted to validate the results of this study.

## MATERIALS AND METHODS

The design and implementation of this meta-analysis were in accordance with the guidelines of the preferred reporting items for systematic reviews and meta-analyses (PRISMA Compliant) statement [33]. All included data were extracted from published studies; no ethical issues were involved too.

### Search strategy

Relevant studies were searched in online databases (such as Science Citation Index, Embase, PubMed, CNKI and Wanfang) to investigate the relationship between the pri-miR-26a-1 rs7372209 C>T polymorphism and the risk of cancer from inception to June 1, 2019. The bibliographies of the relevant reviews and studies that were included were retrospectively assessed to identify more articles. The following search terms and strategy were used (e.g., PubMed database):

#1 pri-miR-26a-1

#2 microRNA-26a

#3 miR-26a

#4 miR-26a-1

#5 rs7372209

#6 #1 OR #2 OR #3 OR #4 OR #5

#7 mutation

#8 variant

#9 polymorphism

#10 #7 OR #8 OR #9

#11 neoplasm

#12 tumor

#13 cancer

#14 #11 OR #12 OR #13

#15 #6 AND #10 AND #14

### Inclusion and exclusion criteria

The inclusion criteria were as follows: (1) case-control studies that investigated the association between the pri-miR-26a-1 rs7372209 C>T polymorphism and the risk of cancer; (2) those with a sufficient number of data on genotype distribution that can be utilized to examine the crude odds ratios (ORs) and 95% confidence intervals (CIs); (3) those written in English and Chinese only; and (4) those in which the largest or most recently sample data were adopted in cases of multiple publications with duplicate or overlapping data on the same theme. Meanwhile, the exclusion criteria were as follows: (1) case series, meta-analyses or reviews; (2) duplicate publications; (3) case-control studies that did not focus on pri-miR-26a-1 rs7372209 C>T locus; (4) unrelated studies; and (5) studies with insufficient data.

### Quality assessment

Two independent authors conducted a quality assessment of all included studies using the modified Newcastle-Ottawa Quality Assessment Scale [34]. Six departments of representativeness of cases, source of controls, Hardy-Weinberg equilibrium (HWE) status in controls, genotyping methods, subjects size and association assessment were involved. The scores ranged from 0 to 11 and studies with more than 8 points were considered of high quality (Table 2).

**Table 2:** Scale for quality evaluation.

**Criteria**		**Score**
**Representativeness of cases**		
Consecutive/randomly selected cases with clearly defined sampling frame		2
Not consecutive/randomly selected case or without clearly defined sampling frame		1
Not described		0
**Source of controls**		
Population-based		2
Hospital-bases or Healthy-bases		1
Not described		0
**Hardy-Weinberg equilibrium status in controls**		
Hardy-Weinberg equilibrium		2
Hardy-Weinberg disequilibrium		1
Not available		0
**Genotyping examination**		
Genotyping done under “blinded” condition and repeated again		2
Genotyping done under “blinded” condition or repeated again		1
Unblinded done or not mentioned and unrepeated		0
**Subjects size**		
Number ≥500		1
Number <500		0
**Association assessment**		
Assess association between genotypes and cancer risk with appropriate statistics and adjustment for confounders		2
Assess association between genotypes and cancer risk with appropriate statistics and without adjustment for confounders		1
Inappropriate statistics used		0

### Data extraction

Two authors (Hu and Jiang) reviewed and extracted the related information from the included studies independently. These data included the first author's name, year of publication, country or region where the study was conducted, control design, race, sample size in the case and control groups, number of data on each genotype, genotyping methods, HWE status in controls, and type of cancer.

### Statistical analysis

The ORs and 95% CIs were calculated to examine the relationship between the pri-miR-26a-1 rs7372209 C>T polymorphism and the risk of cancer. A more scientific statistical method was used in selecting the genetic models. The two pooled logORs of log(CT vs. CC) and log(TT vs. CC) for rs7372209 C>T were calculated first; then, the ratio λ of the two logORs was assessed using the following formula: λ=log(AG vs. AA)/log(GG vs. AA). The genetic model was inferred and calculated using the ratio λ when the value of λ is equal to 0, 0.5, and 1, which correspond to the recessive, co-dominant, and dominant models, respectively. Otherwise, when the ratio λ is <0 or >1, an over dominant model was considered appropriated [35, 36]. Furthermore, a univariate meta-analysis was performed to examine the rest of the genetic models, which included allele contrast, co-dominant, dominant, recessive, and over-dominant models. The heterogeneity among the included studies was examined with the Cochran’s Q test and I^2^ statistical method [37]. The random-effect model was adopted when the I^2^ value exceeded 40%, whereas the fixed-effect model was used then [38, 39]. Subgroup analyses were performed based on the HWE status, race diversity, control design, and type of cancer. Moreover, a meta-regression analysis was conducted to identify the factors contributed to heterogeneity among the studies. Cumulative meta-analyses were conducted to assess the trend of changes in the result. Furthermore, sensitivity analyses were performed to assess the changes in the result. The Egger’s linear regression and Begg’s funnel plots were used to examine potential publication biases [40, 41]. Finally, a trial sequential analysis (TSA) was conducted in selected genetic model. The TSA was conducted with a 5% risk of type I error and a 20% risk of the type II error [42]. STATA version 14.0 (Stata Corporation, College Station, TX, USA) was used in the statistical analysis. A two-sided *P* value<0.05 was considered as statistically significant.

## Supplementary Material

Supplementary Table 1
