# Supplementary material for: Association between the pri-miR-26a-1 rs7372209 C>T polymorphism and cancer susceptibility: multivariate analysis and trial sequential analysis
Source: Aging (Albany NY). 2020 Oct 14;12(19):19060–72. doi: 10.18632/aging.103696 (PMC7732283; doi:10.18632/aging.103696)
Supplement: Supplementary Table 1 [file aging-12-103696-s001..docx]

**Supplementary Table 1. Summary ORs and 95% CI of pri-miR-26a-1 rs7372209 C>T polymorphism and cancer risk.**

|  |  | T vs. C | | | |  | CT vs. CC | | | |  | TT vs. CC | | | |  | CT+TT vs. CC | | | |  | TT vs. CC+CT | | | |  | CC+TT vs. CT | | | |
| --- | --- | --- | --- | --- | --- | --- | --- | --- | --- | --- | --- | --- | --- | --- | --- | --- | --- | --- | --- | --- | --- | --- | --- | --- | --- | --- | --- | --- | --- | --- |
|  | N* | OR | 95% CI | *P* | *I*^2^ |  | OR | 95% CI | *P* | *I*^2^ |  | OR | 95% CI | *P* | *I*^2^ |  | OR | 95% CI | *P* | *I*^2^ |  | OR | 95% CI | *P* | *I*^2^ |  | OR | 95% CI | *P* | *I*^2^ |
| Total | 12 | 0.98 | 0.89-1.07 | 0.60 | 52.5 |  | 0.93 | 0.83-1.06 | 0.28 | 53.5 |  | 1.12 | 0.97-1.28 | 0.13 | 33.3 |  | 0.95 | 0.84-1.06 | 0.37 | 52.9 |  | 1.11 | 0.97-1.27 | 0.11 | 38.5 |  | 1.08 | 0.96-1.22 | 0.20 | 54.4 |
| HWE status |  |  |  |  |  |  |  |  |  |  |  |  |  |  |  |  |  |  |  |  |  |  |  |  |  |  |  |  |  |  |
| Yes | 9 | 1.03 | 0.97-1.09 | 0.37 | 31.8 |  | 1.09 | 0.93-1.01 | 0.89 | 18.8 |  | 1.05 | 0.84-1.31 | 0.68 | 50.2 |  | 1.02 | 0.94-1.10 | 0.64 | 19.3 |  | 1.03 | 0.83-1.29 | 0.78 | 52.2 |  | 1.01 | 0.93-1.09 | 0.84 | 21.5 |
| No | 3 | 0.81 | 0.57-1.15 | 0.24 | 76.6 |  | 0.70 | 0.48-1.01 | 0.06 | 68.1 |  | 1.24 | 0.79-1.93 | 0.35 | 0 |  | 0.72 | 0.49-1.07 | 0.10 | 72.3 |  | 1.35 | 0.87-2.09 | 0.18 | 0 |  | 1.46 | 1.02-2.08 | 0.04 | 66.1 |
| Race |  |  |  |  |  |  |  |  |  |  |  |  |  |  |  |  |  |  |  |  |  |  |  |  |  |  |  |  |  |  |
| Asian | 9 | 1.03 | 0.97-1.10 | 0.32 | 13.5 |  | 0.97 | 0.89-1.05 | 0.43 | 0.0 |  | 1.19 | 1.02-1.38 | 0.03 | 16.0 |  | 1.00 | 0.92-1.08 | 0.96 | 4.4 |  | 1.19 | 1.03-1.38 | 0.02 | 9.0 |  | 1.06 | 0.98-1.15 | 0.15 | 0 |
| Other | 3 | 0.69 | 0.41-1.16 | 0.16 | 80.9 |  | 0.69 | 0.34-1.41 | 0.31 | 87.8 |  | 0.70 | 0.47-1.06 | 0.09 | 0 |  | 0.68 | 0.35-1.30 | 0.24 | 85.9 |  | 0.66 | 0.44-0.98 | 0.04 | 0 |  | 1.43 | 0.68-3.01 | 0.35 | 88.9 |
| Design |  |  |  |  |  |  |  |  |  |  |  |  |  |  |  |  |  |  |  |  |  |  |  |  |  |  |  |  |  |  |
| HB | 7 | 1.04 | 0.96-1.11 | 0.35 | 24.9 |  | 0.97 | 0.88-1.07 | 0.52 | 15.8 |  | 1.21 | 1.01-1.45 | 0.04 | 23.0 |  | 1.00 | 0.91-1.10 | 1.00 | 23.2 |  | 1.22 | 1.02-1.45 | 0.03 | 17.4 |  | 1.06 | 0.96-1.17 | 0.22 | 0 |
| PB | 5 | 0.86 | 0.70-1.06 | 0.16 | 71.4 |  | 0.83 | 0.63-1.11 | 0.21 | 75.8 |  | 0.92 | 0.65-1.29 | 0.62 | 41.4 |  | 0.83 | 0.64-1.09 | 0.18 | 73.8 |  | 0.90 | 0.61-1.32 | 0.58 | 54.8 |  | 1.19 | 0.89-1.59 | 0.24 | 78.0 |
| Type |  |  |  |  |  |  |  |  |  |  |  |  |  |  |  |  |  |  |  |  |  |  |  |  |  |  |  |  |  |  |
| ESCC | 4 | 0.81 | 0.60-1.10 | 0.17 | 77.8 |  | 0.74 | 0.52-1.03 | 0.08 | 74.0 |  | 1.19 | 0.92-1.54 | 0.18 | 0 |  | 0.75 | 0.53-1.07 | 0.11 | 77.0 |  | 1.22 | 0.95-1.56 | 0.12 | 0 |  | 1.37 | 0.99-1.89 | 0.06 | 71.8 |
| LC | 2 | 1.13 | 0.89-1.44 | 0.33 | 60.2 |  | 1.00 | 0.65-1.55 | 1.00 | 76.9 |  | 1.62 | 1.17-2.25 | <0.01 | 0 |  | 1.07 | 0.71-1.62 | 0.75 | 76.4 |  | 1.51 | 1.11-2.06 | 0.01 | 0 |  | 1.07 | 0.74-1.56 | 0.70 | 70.6 |
| CRC | 2 | 1.01 | 0.91-1.12 | 0.90 | 0 |  | 0.93 | 0.81-1.06 | 0.28 | 0 |  | 1.18 | 0.90-1.53 | 0.22 | 0 |  | 0.96 | 0.85-1.09 | 0.55 | 0 |  | 1.22 | 0.94-1.57 | 0.14 | 0 |  | 1.10 | 0.96-1.25 | 0.17 | 0 |

^*^ Numbers of comparisons

HB: Hospital-based PB: Population-based

ESCC: Esophageal Squamous Cell Carcinoma; LC: lung cancer; CRC: colorectal cancer
